# Which Contextual Features Predict Physiological Stress Reactivity in Midlife to Late Life?

**DOI:** 10.1093/geroni/igaa057.1902

**Published:** 2020-12-16

**Authors:** Viktoryia Kalesnikava

**Affiliations:** University of Michigan, Ann Arbor, Michigan, United States

## Abstract

Chronic stress creates vulnerability to adverse mental and physical health outcomes in later life. While claims about the negative effects of stress on health are primarily based on self-report, it is unclear how subjective stress measures (chronic or perceived stress) and other environmental or individual characteristics (neighborhood, social and health behaviors) relate to physiological stress response. This study examines which contextual features contribute to differences in physiological stress reactivity among adults at risk of type II diabetes (Richmond Stress and Sugar Study, n=125, aged 40-70). Psycho-social stress was induced via Trier Social Stress Test. Using advanced selection methods, we simultaneously explore multiple predictors and illustrate how different sets of risk and protective factors contribute to normal or abnormal stress reactivity profiles. Preliminary results suggest that the top five important predictors are education, contact with friends, perceived stress, ruminative coping, and sedentary behavior. Implications for research and targeted interventions are discussed.

